# Supplementary material for: Construction and Validation of a Nomogram Clinical Prediction Model for Predicting Osteoporosis in an Asymptomatic Elderly Population in Beijing
Source: J Clin Med. 2023 Feb 6;12(4):1292. doi: 10.3390/jcm12041292 (PMC9967366; doi:10.3390/jcm12041292)
Supplement: Supplementary file 1 [file jcm-12-01292-s001.zip › Table S1.pdf]

Table S1. Comparison of baseline data for the training and validation groups.

	Training group *N=438*	Validation group *N=146*	p.value
**OP:**			0.923
No osteoporosis	253 (57.8%)	83 (56.8%)	
Osteoporosis	185 (42.2%)	63 (43.2%)	
**Age**	67.2±6.48	66.1±6.31	0.095
**Gender:**			0.789
Female	319 (72.8%)	104 (71.2%)	
Male	119 (27.2%)	42 (28.8%)	
**Manual laborers:**			0.249
No	292 (66.7%)	89 (61.0%)	
Yes	146 (33.3%)	57 (39.0%)	
**Education Level:**			0.950
junior high school	133 (30.4%)	46 (31.5%)	
high school	192 (43.8%)	64 (43.8%)	
undergraduate	113 (25.8%)	36 (24.7%)	
**Height**	160 [156;167]	161 [157;168]	0.460
**Weight**	64.0 [58.0;70.0]	65.0 [59.0;70.0]	0.886
**Waistline**	84.0 [79.0;90.0]	84.0 [79.0;90.0]	0.797
**Smooking:**			0.556
No	388 (88.6%)	126 (86.3%)	
Yes	50 (11.4%)	20 (13.7%)	
**Drinking:**			0.822
No	389 (88.8%)	128 (87.7%)	
Yes	49 (11.2%)	18 (12.3%)	

Notes: Numerical variables for normality were represented as mean ± standard deviation. Non-normal numerical variables were represented by median ± upper and lower quartiles. Categorical variables were represented by frequency and percentage. $p<0.05$ meant the difference was statistically significant.
