# Supplementary material for: Construction and Validation of a Nomogram Clinical Prediction Model for Predicting Osteoporosis in an Asymptomatic Elderly Population in Beijing
Source: J Clin Med. 2023 Feb 6;12(4):1292. doi: 10.3390/jcm12041292 (PMC9967366; doi:10.3390/jcm12041292)
Supplement: Supplementary file 1 [file jcm-12-01292-s001.zip › Table S2.pdf]

Table S2. Interaction effects among various clinical factors.

	Age	Gender	Manualla borers	Education Level II vs I	Education Level III vs I	Height	Weight	Waistline	Smooking	Drinking
**Age**		0.7924	0.1594	0.3519	0.5774	0.5466	0.5354	0.5797	0.8676	0.14
**Gender**			0.4264	0.8687	0.9951	0.882	0.2483	0.291	0.7471	0.6966
**Manualla borers**				0.3474	0.3472	0.5743	0.9451	0.6882	0.1595	0.3773
**Education Level II vs I**										
**Education Level III vs I**										
**Height**				0.6104	0.537		0.2624	0.0064*	0.7421	0.3224
**Weight**				0.5019	0.2152			0.0659	0.4531	0.4587
**Waistline**				0.0368	0.1677				0.6958	0.9956
**Smooking**				0.8305	0.4171					0.7786
**Drinking**				0.956	0.7235					

Notes: Numerical variables were transformed into binary variables, and multi-categorical variables were transformed into dummy variables, and then the interaction effects were analyzed. *$p < 0.05$, which meant the interaction between the two factors was statistically significant.
