# Supplementary material for: Construction and Validation of a Nomogram Clinical Prediction Model for Predicting Osteoporosis in an Asymptomatic Elderly Population in Beijing
Source: J Clin Med. 2023 Feb 6;12(4):1292. doi: 10.3390/jcm12041292 (PMC9967366; doi:10.3390/jcm12041292)
Supplement: Supplementary file 1 [file jcm-12-01292-s001.zip › Table S3.pdf]

Table S3. Univariate and multivariate logistic regression analysis for the training group.

Clinical factors		No osteoporosis (N=253)	Osteoporosis (N=185)	OR (univariable)	OR (multivariable)
**Age**		66.9±6.7	67.5±6.2	1.02 (0.99-1.05, p=.317)	
**Gender**	Male	93 (36.8%)	26 (14.1%)		
	Female	160 (63.2%)	159 (85.9%)	3.55 (2.18-5.79, p<.001)	2.92 (1.21-7.06, **p=.017**)
**Manual laborers**	No	175 (69.2%)	117 (63.2%)		
	Yes	78 (30.8%)	68 (36.8%)	1.30 (0.87-1.95, p=.194)	1.14 (0.71-1.84, p=.590)
**Education Level**	1	49 (19.4%)	84 (45.4%)		
	2	125 (49.4%)	67 (36.2%)	0.31 (0.20-0.50, p<.001)	0.40 (0.24-0.67, **p<.001**)
	3	79 (31.2%)	34 (18.4%)	0.25 (0.15-0.43, p<.001)	0.47 (0.25-0.87, **p=.017**)
**Height**		162 [158;169]	160[155;164]	0.94 (0.91-0.96, p<.001)	1.04 (0.99-1.08, p=.108)
**Weight**		67.0 [60.0;75.0]	60.0 [55.0;65.0]	0.90 (0.88-0.92, p<.001)	0.91 (0.88-0.94, **p<.001**)
**Waistline**		86.0 [80.0;92.0]	80.0 [76.0;86.0]	0.94 (0.92-0.96, p<.001)	0.99 (0.96-1.02, p=.607)
**Smooking**	No	219 (86.6%)	169 (91.4%)		
	Yes	34 (13.4%)	16 (8.6%)	0.61 (0.33-1.14, p=.122)	1.84 (0.68-4.96, p=.231)
**Drinking**	No	217 (85.8%)	172 (93%)		
	Yes	36 (14.2%)	13 (7%)	0.46 (0.23-0.89, p=.020)	1.20 (0.46-3.13, p=.712)

Notes: Univariate logistics regression analysis was performed for all factors. Multivariate logistics regression analysis was performed for statistically significant factors. Odds ratios, confidence intervals, and p-values were shown. $p < 0.05$ meant the difference was statistically significant. Numerical variables for normality were represented as mean ± standard deviation. Non-normal numerical variables were represented by median ± upper and lower quartiles. Categorical variables were represented by frequency and percentage. The “1”, “2” and “3” for “Education Level” stand for “Junior high school”, “High school” and “Undergraduate”. **Abbreviations:** OR, Odds ratios; CI, confidence intervals.
