# Supplementary material for: Construction and Validation of a Nomogram Clinical Prediction Model for Predicting Osteoporosis in an Asymptomatic Elderly Population in Beijing
Source: J Clin Med. 2023 Feb 6;12(4):1292. doi: 10.3390/jcm12041292 (PMC9967366; doi:10.3390/jcm12041292)
Supplement: Supplementary file 1 [file jcm-12-01292-s001.zip › Table S4.pdf]

Table S4. Univariate and multivariate logistic regression analysis for the validation group.

Clinical factors		No osteoporosis (N=83)	Osteoporosis (N=63)	OR (univariable)	OR (multivariable)
**Age**		65.6 ± 6.9	66.8 ± 5.4	1.03 (0.98-1.09, p=.256)	
**Gender**	Male	34 (41%)	8 (12.7%)		
	Female	49 (59%)	55 (87.3%)	4.77 (2.02-11.29, p<.001)	1.35 (0.29-6.39, p=.705)
**Manual laborers**	No	50 (60.2%)	39 (61.9%)		
	Yes	33 (39.8%)	24 (38.1%)	0.93 (0.48-1.83, p=.838)	
**Education Level**	1	18 (21.7%)	28 (44.4%)		
	2	36 (43.4%)	28 (44.4%)	0.50 (0.23-1.08, p=.078)	0.45 (0.19-1.08, p=.075)
	3	29 (34.9%)	7 (11.1%)	0.16 (0.06-0.43, p<.001)	0.21 (0.07-0.66, **p=.008**)
**Height**		163 [160;171]	158[156;162]	0.91 (0.87-0.96, p<.001)	0.99 (0.91-1.07, p=.783)
**Weight**		67.0 [61.0;74.5]	60.0 [54.0;65.0]	0.90 (0.86-0.94, p<.001)	0.93 (0.87-1.00, **p=.035**)
**Waistline**		85.0 [80.0;90.0]	80.0 [76.0;86.5]	0.93 (0.89-0.97, p=.002)	0.98 (0.92-1.04, p=.546)
**Smooking**	No	66 (79.5%)	60 (95.2%)		
	Yes	17 (20.5%)	3 (4.8%)	0.19 (0.05-0.70, p=.012)	0.45 (0.08-2.51, p=.363)
**Drinking**	No	68 (81.9%)	60 (95.2%)		
	Yes	15 (18.1%)	3 (4.8%)	0.23 (0.06-0.82, p=.024)	1.15 (0.22-5.95, p=.871)

Notes: Univariate logistics regression analysis was performed for all factors. Multivariate logistics regression analysis was performed for statistically significant factors. Odds ratios, confidence intervals, and p-values were shown. $p < 0.05$ meant the difference was statistically significant. Numerical variables for normality were represented as mean ± standard deviation. Non-normal numerical variables were represented by median ± upper and lower quartiles. Categorical variables were represented by frequency and percentage. The "1", "2" and "3" for "Education Level" stand for "Junior high school", "High school" and "Undergraduate". **Abbreviations:** OR, Odds ratios; CI, confidence intervals.
