# Supplementary material for: Construction and Validation of a Nomogram Clinical Prediction Model for Predicting Osteoporosis in an Asymptomatic Elderly Population in Beijing
Source: J Clin Med. 2023 Feb 6;12(4):1292. doi: 10.3390/jcm12041292 (PMC9967366; doi:10.3390/jcm12041292)
Supplement: Supplementary file 1 [file jcm-12-01292-s001.zip › Table S5.pdf]

Table S5. Internal validation for training group and validation group.

	Training group		Validation group	
	**AUC[95%CI]**	**C-index[95%CI]**	**AUC[95%CI]**	**C-index[95%CI]**
3 fold cross validation	0.751[0.746,0.756]	0.746[0.741,0.751]	0.750[0.742,0.758]	0.744[0.736,0.752]
5 fold cross validation	0.751[0.745,0.758]	0.746[0.740,0.753]	0.752[0.741,0.764]	0.747[0.735,0.758]
10 fold cross validation	0.750[0.738,0.761]	0.746[0.735, 0.757]	0.750[0.732,0.768]	0.744[0.726,0.763]
Jackknife validation	0.746	0.746	0.738	0.738
Bootstrap validation	0.761[0.759,0.765]	0.756[0.754,0.760]	0.775[0.770,0.780]	0.769[0.764,0.774]

Notes: Internal validation results (AUC and C-index) based on 5 different methods. 3 fold, 5 fold and 10 fold validation with 200 iterations. Jackknife validation with 438 iterations for training group. Jackknife validation with 146 iterations for validation group. Bootstrap validation with 200 iterations. **Abbreviations:** AUC, area under the ROC curve; C-index, concordance index; CI, confidence interval.
